# Halophilic bacteria of salt lakes and saline soils of the Peri-Caspian lowland (Republic of Daghestan) and their biotechnological potential

**DOI:** 10.18699/VJ21.026

**Published:** 2021-03

**Authors:** E.A. Khalilova, S.T. Kotenko, E.A. Islammagomedova, A.A. Abakarova, N.А. Chernyh, D.A. Aliverdiyeva

**Affiliations:** Precaspian Institute of Biological Resources of the Daghestan Federal Research Center of the Russian Academy of Sciences, Makhachkala, Daghestan, Russia; Precaspian Institute of Biological Resources of the Daghestan Federal Research Center of the Russian Academy of Sciences, Makhachkala, Daghestan, Russia; Precaspian Institute of Biological Resources of the Daghestan Federal Research Center of the Russian Academy of Sciences, Makhachkala, Daghestan, Russia; Precaspian Institute of Biological Resources of the Daghestan Federal Research Center of the Russian Academy of Sciences, Makhachkala, Daghestan, Russia; Winogradsky Institute of Microbiology, Federal Research Center “Fundamentals of Biotechnology” of the Russian Academy of Sciences, Moscow, Russia; Precaspian Institute of Biological Resources of the Daghestan Federal Research Center of the Russian Academy of Sciences, Makhachkala, Daghestan, Russia

**Keywords:** bacteria of the genus Halomonas and Virgibacillus, salt lakes, salt marshes soils, biotechnological potential, бактерии, род Halomonas, род Virgibacillus, соленые озера, почвы, солончаки, биотехнологический потенциал

## Abstract

The article presents the results of studying the biodiversity and biotechnological potential of halophilic microorganisms from the thermal highly mineralized Berikey Lake, the salty Lake Tarumovskoye and saline soils of the Peri-Caspian Lowland (Republic of Daghestan). Denitrifying halophilic bacteria of the genus Halomonas and Virgibacillus were identified using microbiological methods and 16S rRNA gene analysis. A new species Halomonas sp. G2 (MW386470) with a similarity of the nucleotide sequences of the 16S rRNA genes is 95 %. Strain G2 is an extreme halophile capable of growing in the range of 5–25 % NaCl (optimum 25 %) and forming a carotenoid pigment. Mesophil, 30–37 °С (optimum 30 °С); neutrophil, pH 6–8 (optimum 7.2–7.4). Strain G2 chemolithotroph; reduces nitrate or nitrite as electron donors; catalase-, amylase-, protease- and β-galactosidase-positive; lipase-, oxidase- and urease-negative. Not able to hydrolyze inositol, indole; produces lysine, gelatin, ectoine; uses citrate and sodium malate as a source of carbon and energy; does not produce ornitin, H2S or acid from d-mannose, sucrose, glycerol, cellobiose, except for lactose and d-glucose. Susceptible to trimethoprim, ciprofloxacin, ofloxacin, kanamycin, vancomycin, rifampicin, cefuroxime, ampicillin, ceftazidime, fosfomycin, clarithromycin, cefepime, cefaclor. The G+C content in DNA is 67.3 %. A distinctive characteristic of the isolate was the production of industrially significant hydrolytic enzymes such as amylase, protease, β-galactosidase, and oxidoreductase (catalase) at a NaCl concentration of 25 % in the medium. Habitat: saline soils on the territory of the Tersko-Kumskaya lowland (Republic of Daghestan, Russia). The rest of the halophilic isolates of H. ventosae G1 (MW386469), H. elongata G3 (MW386471), V. salinarius B2 (MW386472), and V. salinarius B3 (MW386473) had a high degree of similarity (100 %) with the type strains of H. elongata DSM 2581Т and V. salarius DSM 18441Т ; the content of G+C in DNA was 65.8, 66.5, 42.8 and 37.3 %, respectively. The strains had a high biotechnological potential at NaCl concentrations of 5 and 25 % in the medium. The data obtained expanded the understanding of the diversity and ecological significance of denitrifying bacteria in the functioning of arid ecosystems and make it possible to identify strains producing enzymes of industrial importance.

## Abstract (ru)

Приведены результаты изучения биоразнообразия и биотехнологического потенциала галофильных микроорганизмов из термального высокоминерализованного Берикейского озера, соленого Тарумовского озера и солончаковых почв Прикаспийской низменности (Республика Дагестан). С использованием микробиологических методов и метода анализа генов 16S рРНК идентифицированы денитрифицирующие галофильные бактерии родов Halomonas и Virgibacillus. Выявлен новый вид Halomonas sp. G2 (MW386470) с 95 % уровнем сходства нуклеотидных последовательностей генов 16S рРНК. Штамм G2 – экстремальный галофил, способный расти в диапазоне 5–25 % NaCl (оптимум 25 %) и образовывать каротиноидный пигмент. Мезофил (30–37 °С, оптимум 30 °С), нейтрофил (рН 6–8, оптимум 7.2–7.4). Хемолитотроф; редуцирует нитрат или нитрит в качестве доноров электронов; каталазо-, амилазо-, протеазо- и β-галактозидазоположительный; липазо-, оксидазо- и уреазоотрицательный. Не способен гидролизовать инозит, индол; продуцирует лизин, желатин, эктоин; в качестве источника углерода и энергии использует цитрат и малат натрия; не продуцирует орнитин, H2S и кислоту из d-маннозы, сахарозы, глицерина, целлобиозы, кроме лактозы и d-глюкозы. Восприимчив к триметоприму, ципрофлоксацину, офлоксацину, канамицину, ванкомицину, рифампицину, цефуроксиму, ампициллину, цефтазидиму, фосфомицину, кларитромицину, цефепиму, цефаклору. Содержание G+C в ДНК 67.3 %. Отличительной характеристикой изолята являлось продуцирование промышленно значимых гидролитических ферментов, таких как амилаза, протеаза, β-галактозидаза и оксиредуктазы – каталазы при концентрации NaCl в среде 25 %. Местообитание: солончаковые почвы на территории Терско-Кумской низменности (Республика Дагестан, Россия). Остальные галофильные изоляты H. ventosae G1 (MW386469), H. elongata G3 (MW386471), V. salinarius B2 (MW386472) и V. salinarius B3 (MW386473) имели высокую степень сходства (100 %) с типовыми штаммами H. elongata DSM 2581Т и V. salarius DSM 18441Т ; содержание G+C в ДНК составляло 65.8, 66.5, 42.8 и 37.3 % соответственно. Штаммы имели высокий биотехнологический потенциал при концентрации NaCl в среде 5 и 25 %. Полученные данные расширили представление о разнообразии и экологическом значении денитрифицирующих бактерий в функционировании засушливых экосистем и выявлении штаммов, продуцирующих ферменты промышленного значения.

## Introduction

The interest in extremophilic microorganisms is relatively high due to their biological uniqueness. Agreat contribution to the study of natural microbial communities was made by the school of Russian scientists (Zavarzin, 2004; Namsaraev et al., 2010; Bonch-Osmolovskaya, Atomi, 2015). Archaea and highly-specialized bacteria of the genera Alcaligenes, Bacillus, Halobacillus, Virgibacillus, Micrococcus, and Pseudomonas (Wang et al., 2019; Banciu et al., 2020; Begmatov et al., 2020) occupy a dominant place in ecological niches with a high salt content (solar salt works, oceans, seas, hypersaline lakes, saline soils, deserts, plants, saline products) and anthropogenic ecosystems with an increased level of mineralization

The largest ecosystems on the planet are saline and hypersaline environments (Ghosh et al., 2019). Daghestan is a unique natural province of Russia that has a variety of natural landscapes due to the influence of tectonic processes, the erosive activity of flowing waters, transgressive and regressive dynamics of the Caspian Sea, and arid climate. There are a number of works on the study of microbial communities of various ecological niches of the region: lithotrophic sulfur-oxidizing representatives of sulfide sources, hydrocarbon-oxidizing bacteria of the geothermal source of the Kizlyar field (Chernousova et al., 2008; Gridneva et al., 2009; Khalilova et al., 2014).

The highly mineralized lakes of the Tersko-Kumskaya lowland with high salinity form conditions for the existence of halophilic bacteria. Microorganisms from extreme habitats are the producers of valuable industrially important enzymes, antibiotics; they can participate in soil biodegradation, and are highly resistant to contamination by foreign microflora (Corral et al., 2020).

The study considers the spatial distribution of halophilic microbial communities of halophyte plants, saline soils, and highly mineralized lakes in arid regions of the Peri-Caspian Lowland (Khalilova et al., 2017, 2020). Chemoorganoheterotrophic bacteria of the genera Virgibacillus, Bacillus, Halomonas and Salimicrobium from the phylum Firmicutes and Proteobacteria have proved to be the main components of the microbial flora of the Tersko-Kumskaya and TerskoSulakskaya provinces. A major correlation was revealed between isolated microbial communities and concentrations of chemicals Na, K, Ca, Mg, Cl, Cu, Sr, SO4, Cl, and HCO3, as one of the main regulators of microbiological activity in soils and lakes.

The aim of the paper is a molecular taxonomic study of isolated halophilic bacteria and their biotechnological potential.

##  Materials and methods

**The objects of research** are natural microbial communities of saline reservoirs and soils in the territory of the PeriCaspian Lowland of the Republic of Daghestan (Khalilova et al., 2020) (Table 1). Samples were taken in July–September 2014.

**Table 1.**
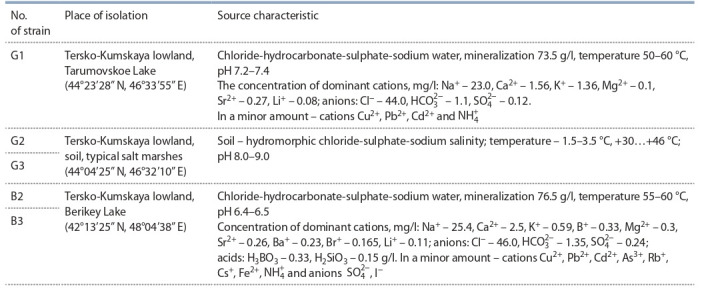
Strains of halophilic bacteria and sources of sampling

**Cultivation.** For the cultivation of halophilic bacteria, a modified medium of the following composition (g/l) was used: bacto yeast extract – 10.00, Na3C6H5O7 ∙5.5H2O – 3.0, NaCl – 50, 100, 250, KCl – 2.0, MgSO4 ∙ 7H2O – 20.0, glycerin – 4.0 (RF Pat. RU 2115722, 1998; RF Pat. RU 2323226, 2008). Bacto-peptone (Difco, Spain) – 5 g/l was used as a substrate; the pH of the medium was adjusted to 7.2–7.4 by 1N HCl or 4M KOH (Russia) using a Hanna Instrumentals pH 211 pH meter (Germany). The cultures were incubated in a Binder-115 microbiological incubator (USA) at an operating temperature of (30–37)±1 °C for 3–20 days.

**Morphology** of bacterial cells (cell morphology, motility, the presence of spore formation) was studied using a light microscope CX21 FS1 (Olympus, Japan) and the PowerShot A640 digital camera (Canon, Japan) at a working magnification of ×600.

**Ecological and physiological characteristics of growth** (temperature, pH, salinity). Effect of NaCl concentration (0, 5, 10, 15, 25 %, weight/volume) in an amount of 2 % of the medium volume for cell growth in liquid and solid media was determined at 30–37 °C in the Binder-115 incubator (USA). The growth was monitored at 24-hour intervals for 7 days by measuring turbidity using a Genesys-20 spectrophotometer (Thermo Spectronic, USA). The effect of temperature (30 and 37 °C) on the growth rate was determined by cultivation under the same conditions.

**The use of growth substrates** (assimilation of organic acids, the formation of acid from carbohydrates, reduction of nitrates to nitrites) was studied using standard methods (Gordon, Smith, 1953; Holt et al., 1997; Netrusov et al., 2005).

**The use of electron acceptors.** The ability to use nitrate as an electron acceptor was determined using the BD BBL Taxo Differentiation Discs Nitrate (Becton Dickinson and Company, Australia), in compliance with the manufacturer’s instructions. The discs were impregnated with a solution containing 40 % potassium nitrate and 0.1 % sodium molybdate. The reduction of nitrate to nitrite was detected by the addition of sulfanilic acid and N,N-dimethyl-α-naphthylamine, which reacts with nitrite to form a red-colored substance-n-sulfobenzenazo-α-naphthylamine (positive result). In the absence of a change in color after the addition of reagents (negative result), zinc dust was added to determine the presence of unrecoverable nitrate or products other than nitrite

**Determination of enzymatic activity.** Hydrolase-producing bacteria were screened for amylase, proteinase, β-galactosidase, lactase, lipase, urease, and oxidoreductases (catalases, oxidases) on dishes of starch, tributyrin, and gelatin agar, depending on the concentrations of NaCl.

Amylase activity – on the elective medium (1.0 % starch, 0.5 % peptone, 0.3 % yeast extract, 1.0 % NaCl). The isolates were incubated at 45 °C for 24–36 hours, tested with Lugol solution (10.0 g potassium iodide, 5 g iodine, 100 ml distilled water). Potential amylase producers were selected based on the ratio of the clearance zone diameter to the colony diameter. Protease was determined on media with agar and 10 % skimmed milk; β-galactosidase (lactase) – using indicator disks impregnated with a special reagent-ortho-nitrophenylβ-d-galactopyranoside (Conda, Spain); urease – using CLO test (Kimberly-Clark, USA); lipase – tested on dishes with 1 % tributyrin. Isolates showing clear zones of tributyrin hydrolysis were identified as lipase-producing bacteria.

Determination of oxidoreductases: catalase – using 3 % H2O2 as a substrate in the medium for 24–48 hours, oxidase – by Kovac’s method (Steel, 1961). All screening tests for enzymatic activity were performed in three repetitions. The bacteria were incubated at 37 °C for seven days.

**Antibiotic resistance** (trimethoprim, ciprofloxacin, ofloxacin, kanamycin, vancomycin, rifampicin, cefuroxime, ampicillin, ceftazidime; fosfomycin, clarithromycin, cefepime, cefaclor) was determined by the intensity of bacterial growth on the basic agarized medium “B” in Petri dishes using standard disks “Indicator paper systems for identifying microorganisms” of NPO Microgen of Natsimbio Holding (Russia) with 10–30 μg of antimicrobial agent (Baumann P., Baumann L., 1986).

**G+C content and phylogenetic analysis.** Genomic DNA was isolated according to Marmur (1961) and Thomas (Thomas et al., 1997) methods. The DNA nucleotide composition was determined by thermal denaturation (0.5 °C∙min–1) using a Cary-100 Bio UV-VIS spectrophotometer (Varian, Australia). The GC content in the composition of DNA – according to the method (Owen et al., 1969). Escherichia coli K-12 DNA (51.7 %) was used as the standard.

For phylogenetic analysis, the DNA was isolated from samples using the modified Birnboim–Doly alkaline DNA method (Birnboim, Doly, 1979) and the Wizard technology of Promega (USA) (Bulygina et al., 2002). The concentration of the resulting DNA sample when using this method was 30–50 μg /ml. RNA in the resulting preparation is present in trace amounts (less than 1 %, according to the data of electrophoretic analysis, which are not presented).

For polymerase chain reaction (PCR) and further sequencing of PCR fragments of the 16S rRNA gene for each of the studied samples, universal primer systems were used to detect both eubacteria (11f-1492r) (Lane, 1991) and archaea (8fa-A915R) (Kolganova et al., 2002). The volume of the amplification mixture was 50 μl with the following composition: 1× buffer of BioTaq DNA polymerase (17 mM (NH4)2SO4, 67 mM Tris-HCl, pH 8.8, 2 mM MgCl2); 12.5 nmol of each dNTP, 50 ng of the DNA matrix; 5 pmol of the corresponding primers and 3 units of BioTaq DNA polymerase (Dialat LTD, Russia). The temperature-time profile of the PCR was as follows: the first cycle – 94 °C× 9 min, 55 °C×1 min, 72 °C×2 min; the next 30 cycles – 94 °C×1 min, 55 °C×1 min, 72 °C×2 min; the final cycle – 72 °C×7 min. The PCR products were analyzed by electrophoresis in 2 % agarose gel at an electric field strength of 6 V/cm. Isolation and purification of PCR products were carried out from low-melting agarose using a set of reagents by WizardPCRPreps (Promega, USA), according to the manufacturer’s recommendations.

Sequencing of PCR products was carried out at the Center of “Bioengineering” of the Russian Academy of Sciences, Moscow, by the method of (Sanger et al., 1977) using a set of BigDyeTerminatorv.3.1 reagents on the ABIPRIZM 3730 genetic analyzer (Applied Biosystems, Inc., USA). Standard primers were used for sequencing (Camacho et al., 2009).

**Analysis of 16S rRNA sequences.** The primary analysis of the similarity of the nucleotide sequences of the 16S rRNA genes of the studied strains was performed using the BLAST program on the following web-site: https://blast.ncbi.nlm.nih.gov (Van de Peer, De Wachter, 1994).

The 16S rRNA gene sequences of all studied strains are deposited in GenBank: G1 – MW386469, G2 – MW386470, G3 – MW386471, B2 – MW386472, B3 – MW386473.

## Results and discussion

Strains of halophilic bacteria G1, G2, G3, B2 and B3, isolated from salt lakes and salt marshes of the TerskoKumskaya and Tersko-Sulakskaya lowlands, grew at a temperature of 30–37 °C and pH 6.4–7.4. The cultures showed steady growth in the agarized elective medium in the presence of 5–25 % NaCl with an optimum of 5, 10, 25 %, which indicated that they belonged to moderate and extreme halophiles following the known classification (Kushner, Kamekura, 1988).

**Fig. 1.**
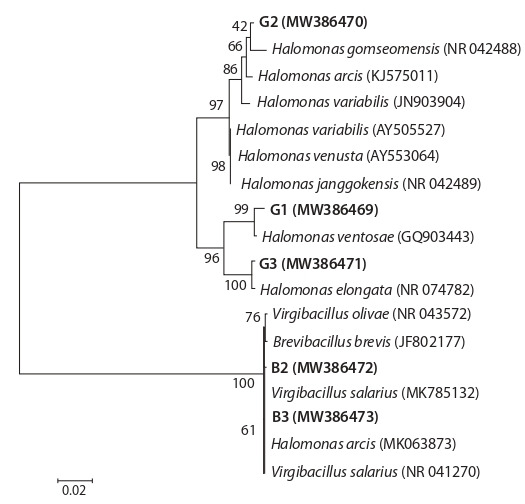
Phylogenetic tree constructed using the Maximum Likelihood method based on the Tamura–Nei model (Tamura, Nei, 1993) and MEGA 6 (Tamura et al., 2013). A total of 18 sequences with a minimum length of 1381 nucleotides were used. Bar corresponds to two substitutions per 100 nucleotides. The Bootstrap values (500 repeats) are shown next to the tree branches.

The analysis of the 16S rRNA gene sequence allowed us to determine their phylogenetic position. The 16S rRNA gene sequences of the new halophilic strains were analyzed and compared with the 16S rRNA sequences of the validly described bacterial species. The analysis has shown that the new isolates belong to two genera of bacteria containing halophilic microorganisms Halomonas and Virgibacillus (Table 2, Fig. 1). Table 2 and Figure 1 demonstrate that the G2 strain represents a new species in the genus Halomonas. The H. ventosae G1 (MW386469) and H. elongata G3 (MW386471) strains seem to belong to the species H. ventosae and H. elongata, respectively, while the V. salinarius B2 (MW386472) and V.salinarius B3 (MW386473) strains belong to the group of species related to V. salinarius.

**Table 2.**
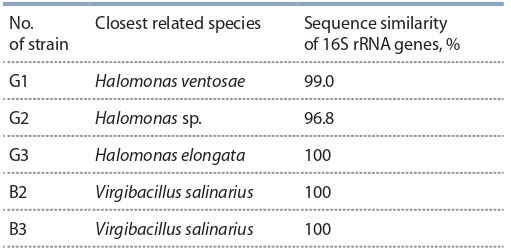
The percentage of similarity of the 16S rRNA gene of halophilic bacteria isolated from salt lakes and salt marshes of the Peri-Caspian Lowland with the most closely related species

**Characteristics of the Halomonas sp. G2 strain**

The main object of further research is the strain Halomonas sp. G2. The GC content in the G2 strain DNA was 67.3 %.

**Fig. 2.**
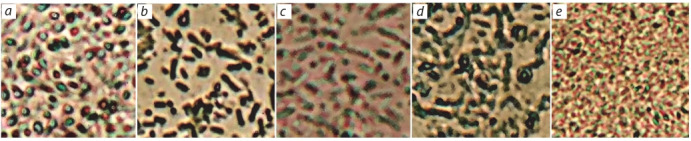
Cell morphology of strains from water and soil samples: a – V. salinarius B2; b – V. salinarius B3; c – H. elongata G1; d – Halomonas sp. G2; e – H. elongata G3. Light microscopy, magnification ×600.

**Fig. 3.**
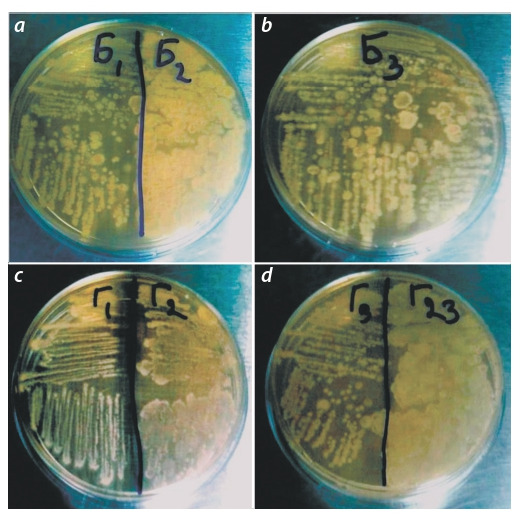
Growth of isolated pure bacterial cultures on MPA: a – V. salinarius B2; b – V. salinarius B3; c – H. elongata G1, Halomonas sp. G2; d – H. elongata G3.

**Morphology of cells and colonies.** Rod-shaped, gramnegative, mobile bacillus of the size of 0.8–1.0×1.5–3.0 µm. The cells were observed singly, in pairs, or in short chains (Fig. 2, d ). Cell mobility was ensured by one or two lateral flagella located on one side of the cell, forming endospores. On an elective solid medium, the strain formed an active growth of colonies of a round shape with a wavy edge, yellow and pale-yellow color with a gloss. With an increase in the concentration of NaCl in the culture medium, a bright carotenoid pigment appeared in the beige-colored colonies. On meat-peptone agar (MPA) – small colonies located close to each other in a chain, rounded shape with a wavy edge, turning into a solid growth; smooth, shiny, light beige with a pinkish tinge. In all variants, a smearing consistency was observed (Fig. 3, c).

**Physiology of strain growth** (temperature, pH, salinity). When determining the optimal growth parameters, the G2 strain was assigned to mesophiles (30 to 37 °C, 30 °C optimum) and moderate alkalophiles (pH 6–8, 7.2–7.4 optimum). As a representative of the genus Halomonas, it can grow in a wide range of NaCl concentrations from 10 to 25 % with an optimum of 25 %; an extreme halophile

**The substrates and electron acceptors used. Oxygen ratio.** The G2 strain is capable of denitrification by using nitrates as an electron acceptor, reducing them to nitrites.

The differentiating characteristics of the G2 strain are presented in Table 3. The strain had a positive reaction to lysine, gelatin, ectoine, lactose, and d-glucose; utilized citrate and Na malonate. Tests for β-galactosidase, amylase, protease, and catalase are positive; for oxidase, lipase, and urease – negative. Growth does not occur under anaerobic conditions.

**Table 3.**
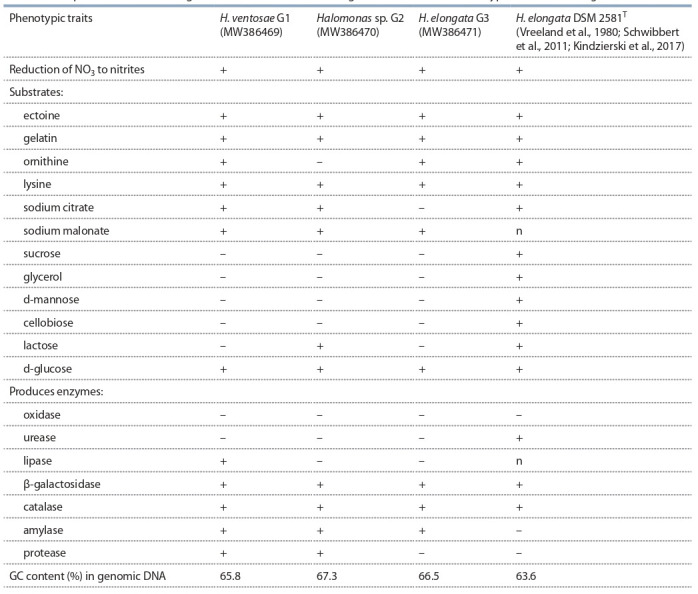
Comparative differentiating features of new strains of the genus Halomonas and the type strain of H. elongata DSM 2581Т Notе. “+” – positive; “–” – negative; “n” – not studied.

**Antibiotic sensitivity.** The G2 culture differed in sensitivity to trimethoprim of the sulfanilamide group; fluoroquinolones of the 1st and 2nd generations (ciprofloxacin, ofloxacin, kanamycin); vancomycin of the macrolide group; rifamycins of the rifampin group; cefuroxime and ampicillin of the penicillin group; antibiotics of the 3rd generation cephalosporins of the macrolides (ceftazidime, fosfomycin and clarithromycin); antibiotics of the 4th generation cephalosporins (cefepime, cefaclor).

**Characteristics of isolates G1, G3, B2, B3**

Rod-shaped, mobile cells of strains G1, G3 have the following sizes: 0.6–0.8 × 1.6–1.9 µm (G1), 0.7–1.0 × 1.5– 2.5 µm (G3) (see Fig. 2, c, e). Single cells and chains of them were observed. Mobility was provided by flagella located on one side of the cell. The cells of the B2 and B3 strains were mobile, in the form of rods, with sizes: 0.5–0.7×1.0–2.5 µm (B2) and 0.2–0.7×1.0–5.0 µm (B3); formed endospores. The biomass of isolated strains on the MPA medium is represented by a chain of colonies located one after another, differing in shape, color, size, pigment and morphology (see Fig. 3, a, b). On an elective agar medium with 5–25 % NaCl (G1, G3) and 5–10 % NaCl (B2, B3), the cultures formed colonies with lipochromic pigment.

The results of phylogenetic analysis of 16S rRNA gene sequences indicated that the closest type strains (100 %) for G1 and G3 are the type strain of H. elongata DSM 2581T, and for B2 and B3 – V. salarius DSM 18441T. On the dendrogram, the cultures formed a common cluster with the typical strains, making it possible to classify isolated cultures as these species. Related cultures for G1 and G3 are H. ventosae GQ903443, H. elongata NR 074782; for B2 and B3 – V. salarius MK785132, B. brevis JF802177, V. olivae NR 043572, H. arcis MK063873, V. salarius NR 041270, which combined the typical features of moderate and extreme halophiles.

The distinctive characteristics differentiating the cultures of H. ventosae G1 and H. elongata G3 were: optimum growth at 5–25 % NaCl vs. 32 %; pH 7.2–7.4 vs. 7–9; no utilization of sucrose, glycerol, d-mannose, cellobiose, lactose, and production of urease, oxidase, and protease (except G3) at a concentration of 5–25 % NaCl in the medium (see Table 3). At the same time, such traits for V.salarius B2 and B3 strains in comparison with the typical V. salarius DSM 18441T were: no need for d-mannose, the ability to produce amylase, protease, and β-galactosidase enzymes at a concentration of 5–10 % NaCl in the medium (Table 4).

**Table 4.**
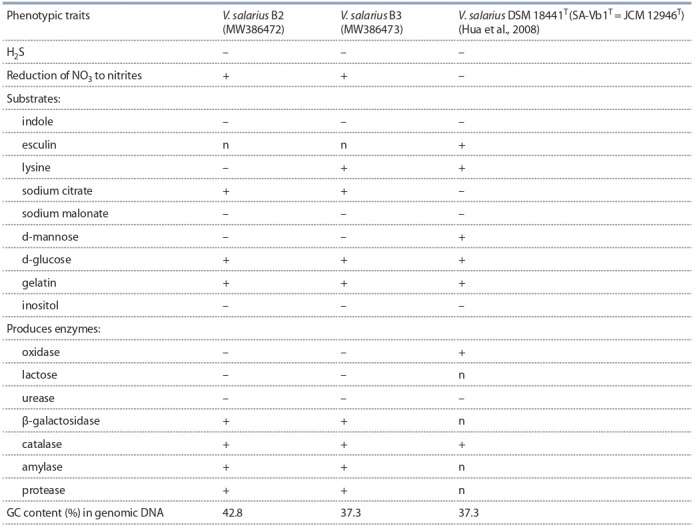
Comparative differentiating features of halophilic strains of V. salarius B2 and B3 with the typical V. salarius DSM 18441T Notе. “+” – positive; “–” – negative; “n” – not studied

**Characteristics of genera Halomonas and Virgibacillus**

Thus, based on phenotypic and genetic studies, we provide a brief description of the strain of the new species Halomonas sp. G2 and halophilic strains of H. ventosa G1, H. elongata G3, V. salinarius B2, V. salinarius B3.

Currently, the genus Halomonas includes 91 species, where H. elongata acts as the type species (http://www. bacterio.cict.fr/h/halomonas.html). The Halomonadaceae family was first described in 1988 by combining moderately halophilic and marine bacteria of the genera Deleya and Halomonas (Franzmann et al., 1988). Over the past three decades, many species have been assigned to the genus Halomonas, the domain Bacteria, the phylum Proteobacteria, the class Gammaproteobacteria, the order Oceanospirillales, and the family Halomonadaceae; however, at the time of writing, 7 species have been reclassified. Representatives of the genus – gram-negative, facultative anaerobes, aerobes, prototrophs, mesophiles, denitrifying, produce exopolysaccharides; they mainly utilize oxygen, nitrate or nitrite as an electron acceptor; under conditions of saline stress, they synthesize ectoine, which protects cells from adverse environmental influences (Schwibbert et al., 2011).

The genus Virgibacillus was created as a result of the reclassification of Bacillus pantothenticus, Virgibacillus pantothenticus and, subsequently, established as a species of Virgibacillus (Heyndrickx et al., 1998; Heyrman et al., 2003). At the moment, the genus consists of 27 species, representatives of which are gram-positive, obligate aerobes or facultative anaerobes, moderate halophiles, chemotaxonomic; the main fatty acid is C15:0 (Lee et al., 2012).

**Description of the strain of the new species Halomonas sp. G2.** The G2 strain cells are encapsulated, motile, aerobic, gram-negative bacillus 0.8–1.0 × 1.5–3.0 µm; observed singly or in a chain of 2 to 4 interlocked cells. The G2 strain is an extreme halophile, capable of growing in the range of 10–25 % NaCl (25 % optimum) and forming a carotenoid pigment. On an elective solid medium with 25 % NaCl, it forms colonies of a round shape with a wavy edge, beige color with a gloss; forms areas of bright carotenoid pigment. The strain grows on meat-peptone broth. The species is mesophile, the temperature range is 30–37 °C (30 °C optimum). Neutrophil, pH 6–8 (7.2–7.4 optimum). Denitrifying strain; chemolithotrophic; reduces nitrate or nitrite as electron donors; catalase-, amylase-, protease-, and β-galactosidase-positive; lipase-, oxidase-, and urease-negative. Unable to hydrolyze inositol, indole; produces lysine, gelatin, ectoine; uses sodium citrate and malate as a source of carbon and energy; does not produce ornithine; H2 S and acid from d-mannose, sucrose, glycerol, cellobiose, except lactose and d-glucose. Susceptible to trimethoprim, ciprofloxacin, ofloxacin, kanamycin, vancomycin, rifampicin, cefuroxime, ampicillin, ceftazidime, fosfomycin, clarithromycin, cefepime, cefaclor. The GC content in DNA is 67.3 %.

Based on its physiological, biochemical, and phylogenetic properties, the G2 strain represents a new species, called Halomonassp. G2. Adistinctive characteristic of the isolate is the production of hydrolytic enzymes protease, amylase, β-galactosidase, and oxy-reductase-catalase at a concentration of 25 % NaCl in the medium.

Habitat: soil (typical saltmarsh) on the territory of the Tersko-Kumskaya lowland (Republic of Daghestan, Russia).

**Tersko-Kumskaya lowland (Republic of Daghestan, Russia). Description of the strains Halomonas ventosae G1 (MW386469), and Halomonas elongata G3 (MW386471).** Halomonas G1 and G3 strains are aerobes, gram-negative, denitrifying; mesophiles, prototrophs, chemolithotrophs, and extreme halophiles (5 to 25 % NaCl). Unable to hydrolyze inositol; produce lysine, ornithine, gelatin, ectoine; reduce nitrate or nitrite as electron donors; utilize citrate (excluding G3) and sodium malonate as a source of carbon and energy; do not produce H2S and acid from d-mannose, sucrose, glycerol, cellobiose, except for d-glucose. The GC content in the DNA for G1 and G3 are 65.8 and 66.5 %, respectively. On the basis of phenotypic and genotypic characteristics, isolated bacteria are classified as H. ventosae G1 (MW386469), and H. elongata G3 (MW386471).

Habitat: saline soils (Tarumovsky district, Kochubey biosphere station) and Lake Tarumovskoe on the territory of the Tersko-Kumskaya lowland (Republic of Daghestan, Russia). The type strain of H. elongata DSM 2581Т was isolated from equipment for extraction of salt from brine (Netherlands Antilles, southern island of Bonaire).

**Description of V. salinarius B2 (MW386472) and B3 (MW386473) strains.** The strains are gram-positive; mesophiles, neutrophils, chemolithotrophs, moderate halophiles (optimum 5, 10 % NaCl). The cultures are unable to hydrolyze inositol, sodium malonate; they do not produce lysine (except for B3), indole, H2S and acid from d-mannose, sucrose, except for d-glucose, reduce nitrate to nitrite and are capable of utilizing the polypeptide substrate gelatin and sodium citrate as a carbon source. The GC content in the DNA of strains B2 andB3 was 42.8 and 37.3 %, respectively. Based on the phenotypic and genotypic characteristics, the isolated cultures have been classified as V. salinarius B2 (MW386472) and V. salinarius B3 (MW386473) strains.

Habitat: waters of technogenic, highly mineralized Berikey Lake (Derbent region, Republic of Daghestan, Russia). The type strain Virgibacillus salarius DSM 18441Т isolated from the salt crust of the Gharsah Salt Lake in Shatt al Gharsah (Sahara) in Tunisia (Hua et al., 2008).

**Biotechnological potential of halophilic microorganisms**

Halophilic bacteria are increasingly being studied for their biotechnological potential for the production of biochemically active and resistant enzymes to alkaline pH, high temperature and salt concentration (Di Donato et al., 2019; Liu et al., 2019). These multifaceted properties are useful for a variety of industries (Delgado-Garcia et al., 2012), such as fermented food, textiles, pharmaceuticals, cosmetics, and leather production (De Lourdes Moreno et al., 2013). Most producers of extracellular hydrolytic enzymes lipase, amylase, protease, inulinase, xylanase, cellulase, DNase, and pectinase are halophilic bacteria, including strains of the genera Halomonas and Virgibacillus (Cira-Chávez et al., 2018; Liu et al., 2019; Kaitouni et al., 2020; Varrella et al., 2020).

Isolation of natural strains in these studies made it possible to discover a new species Halomonas sp. G2 (MW386470) and new strains Halomonas G1 (MW386469) and G3 (MW386471), Virgibacillus B2 (MW386472) and B3 (MW386473), capable of producing hydrolytic enzymes (amylase, protease, lactase, lipase, urease, β-galactosidase) and oxidoreductase (catalase, oxidase).

## Conclusion

The study confirms the biotechnological and scientific importance of halophilic denitrifying bacteria inhabiting the extremophilic ecological niches of the Peri-Caspian Lowland in the Republic of Daghestan. The strains of bacteria of the genera Halomonas and Virgibacillus isolated proved to be not strictly confined to the salt lakes and soils of the Peri-Caspian Lowland (Republic of Daghestan, Russia), having a wide distribution area, including the ecological niches of Bonaire Island (Netherlands Antilles) and Tunisia. Isolation and study of natural strains have revealed a new species Halomonassp. G2 that complement the collection of the already known strains – producers of industrially useful enzymes such as amylase, protease, lactase, lipase, urease, β-galactosidase, catalase, and oxidase.

## Conflict of interest

The authors declare no conflict of interest.
